# Conducting multicenter trials through the trial innovation network comprehensive consultation

**DOI:** 10.1016/j.cct.2026.108334

**Published:** 2026-05-03

**Authors:** Daniel F. Hanley, Karen Lane, Nichol McBee, Lindsay M. Eyzaguirre, W. Andrew Mould, Elizabeth Holthouse, Jessica F. Baird, Jonathan D. Casey, Consuelo H. Wilkins, Salina P. Waddy, Ken L. Wiley, Sara Hassani, Meghan Hildreth, Angeline Nanni, Terri L. Edwards, Dixie D. Thompson, Mary Stroud, Emily Serdoz, Nan Kennedy, Sarah J. Nelson, Michelle Jones, Leslie R. Boone, Colleen Lawrence, Sarah K. Cook, Tiffany Chen, Jodie Cohen, Natalie Dilts, Alex C. Cheng, Randall W. Grout, Edgar R. Miller, James F. Casella, Daniel K. Benjamin, Harry P. Selker, Marisha E. Palm, Lori Poole, Jeri S. Burr, Daniel E. Ford, Gordon R. Bernard, Paul A. Harris

**Affiliations:** a Department of Neurology, BIOS Clinical Trial Coordinating Center, Trial Innovation Center, Johns Hopkins University School of Medicine, Baltimore, MD, USA; b Johns Hopkins Institute for Clinical and Translational Research, Baltimore, MD, USA; c Vanderbilt Institute for Clinical and Translational Research, Nashville, TN, USA; d Department of Medicine, Vanderbilt University Medical Center, Nashville, TN, USA; e Department of Internal Medicine, Meharry Medical College, Nashville, TN, USA; f National Center for Advancing Translational Sciences, National Institutes of Health, Rockville, MD, USA; g University of Utah Health, Salt Lake City, UT, USA; h Utah Clinical & Translational Science Institute, Salt Lake City, UT, USA; i Department of Biomedical Informatics, Vanderbilt University Medical Center, Nashville, TN, USA; j Indiana University School of Medicine, Indianapolis, IN, USA; k The Regenstrief Institute, Inc., Indianapolis, IN, USA; l Johns Hopkins University School of Medicine, Baltimore, MD, USA; m Duke University School of Medicine, Durham, NC, USA; n Institute for Clinical Research and Health Policy Studies, Tufts Medical Center, Boston, MA, USA; o Tufts Clinical and Translational Science Institute, Tufts University, Boston, MA, USA; p Duke Clinical Research Institute, Durham, NC, USA

**Keywords:** Clinical trials, Informative research, Medicine, Trial innovation network, Research ethics

## Abstract

Effective design and execution of multicenter clinical trials remain critical yet challenging for advancing clinical care. The Trial Innovation Network (TIN) Comprehensive Consultation process was established to improve the feasibility and success of investigator-initiated trial proposals by offering structured, multidisciplinary support across 60+ Clinical and Translational Science Award (CTSA) institutions. We report outcomes from 75 trial proposals that underwent Comprehensive Consultation between 2016 and 2024. The process follows a phased Assess–Design–Compose model that integrates expertise in trial design, operations, recruitment, and informatics to generate robust, fundable grant applications. Here we demonstrate 66% of our submitted grants receive funding. This consultation framework demonstrates a scalable, high-impact approach to enhancing the rigor, efficiency, and success of multicenter clinical trials and offers a model for national and global networks seeking to improve clinical research translation.

## 1. Introduction

Well-planned, informative multicenter clinical trials are essential for advancing clinical practice. An effective trial design that includes a prospectively defined analysis plan targeting an important clinical question, adequate resources, and community equipoise can drive feasibility and produce meaningful, generalizable results [1]. Too often, one or more of these conditions are overlooked, and trial proposals fail scientific review. Historically, the success rate for new NIH research project grant awards, including trials, has been low, ranging from 20.7% in 2022 to 21.3% in 2023 [2]. The Trial Innovation Network (TIN) Comprehensive Consultation process was established in 2016 to address common barriers to multicenter proposal development and execution, accelerating findings into clinical practice [1,3-5]. This research letter describes the TIN Comprehensive Consultation process, which draws on interdisciplinary teams of experts and a network of 60+ Clinical and Translational Science Award (CTSA) institutions to develop study proposals, into innovative, rigorous grant applications and eventual high-quality clinical trials.

## 2. Methods

In order to initiate a Comprehensive Consultation, consultees submit a proposal intake form to the TIN for advice and collaboration and undergo an Initial Consultation [6]. Consultees are investigators who are seeking expert advice and resources for multicenter clinical trial study design including developing proposals into protocols, enhancing study operations, and improving recruitment and retention. Consultees can range from post-doctoral fellows to professors at institutions of varying funding levels [7]. After the Initial Consultation, the consultee can then choose to proceed with developing a comprehensive proposal in partnership with the TIN. Each Initial Consultation must reach consensus approval from a TIN committee called the Proposal Assessment Team (PAT), and investigators (consultees) must wish to engage the operational support of the TIN, then a Comprehensive Consultation is initiated to plan a trial. Notably, approval from the PAT reflects an assessment of project readiness, feasibility, and alignment with TIN capabilities, rather than a determination of scientific quality or likelihood of funding. The Comprehensive Consultation leverages the expertise of Trial Innovation Centers (TICs) and a Recruitment Innovation Center (RIC) [8] to strengthen trial design, including primary hypothesis, feasibility, recruitment and retention planning, statistical analysis, clinical, data management, operational plans, and results reporting.

**Fig. 1.**
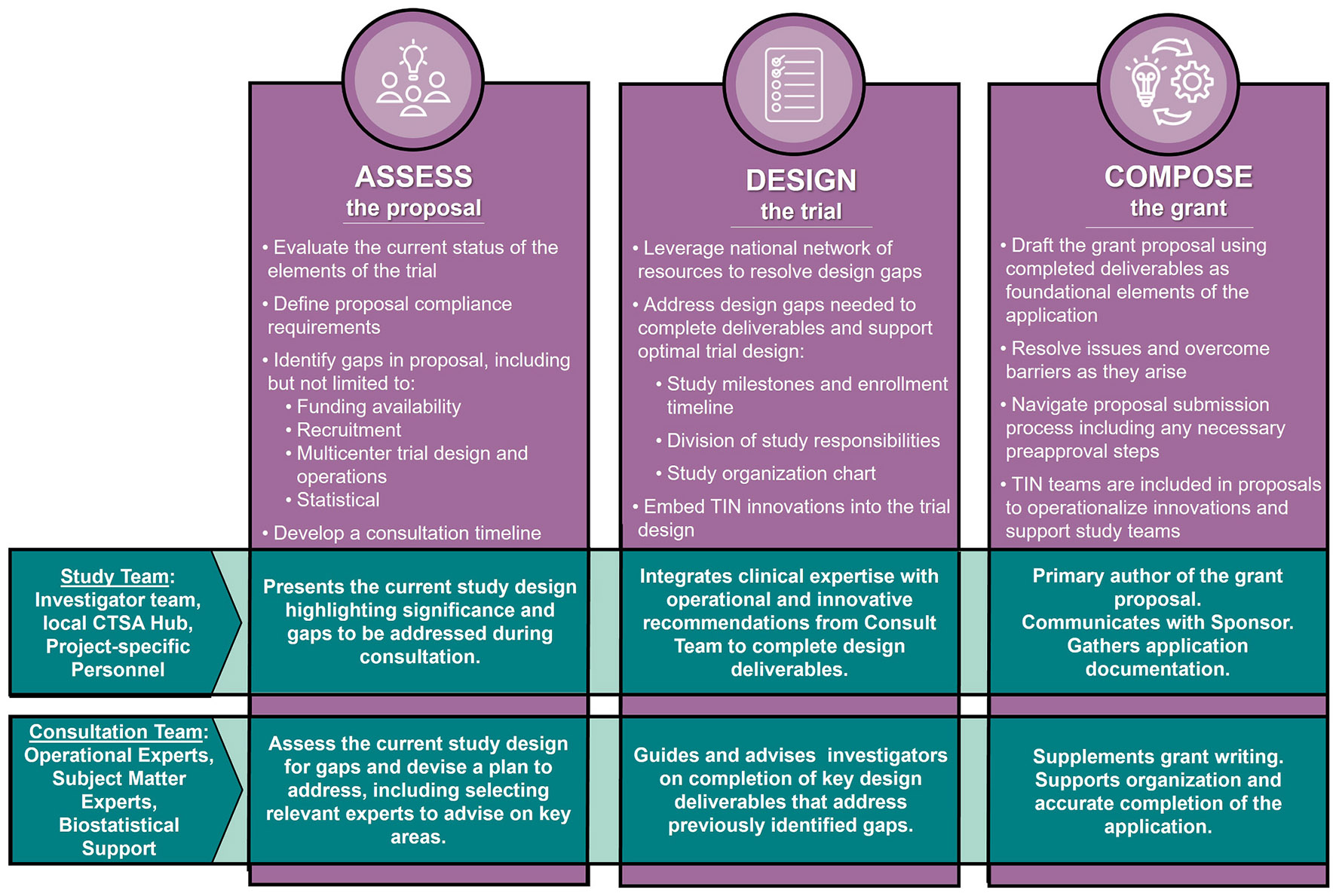
Workflow of the Comprehensive Consultation Process. During the comprehensive consultation, the TIN Consultation Team and the Study Team work together to assess, design, operate, and ultimately submit a compelling grant proposal. Throughout the process, the multidisciplinary consultation team—including subject matter experts, trial operation experts, and biostatisticians—work collaboratively with the study team, ensuring that a trial is clinically important, well-supported, and methodologically rigorous.

The Comprehensive Consultation follows an Assess-Design-Compose approach (Fig. 1). During the Assess Phase, investigators meet with TIC/RIC experts to consider trial design needs for an informative trial, identify funding, and agree on solutions to enhance trial informativeness around the requirements of a trial sponsor for trial design and conduct. The Design Phase focuses on best-practice deliverables, such as the study protocol, statistical analysis plan, budget, timelines, and recruitment and retention strategy. The process leverages the CTSA's national network of resources, and electronic health record (EHR) data to inform study design, and plan outreach to potential TIN sites for selection. TIN innovations are embedded into trial design and operations [5,9-11]. Each consultation is evaluated in all planning domains. In the Compose Phase, deliverables become the foundational blocks of a grant proposal, incorporating innovative and impactful multicenter trial elements as well as RIC/TIC expert recommendations and tools into a final application for submission.

## 3. Results

**Fig. 2.**
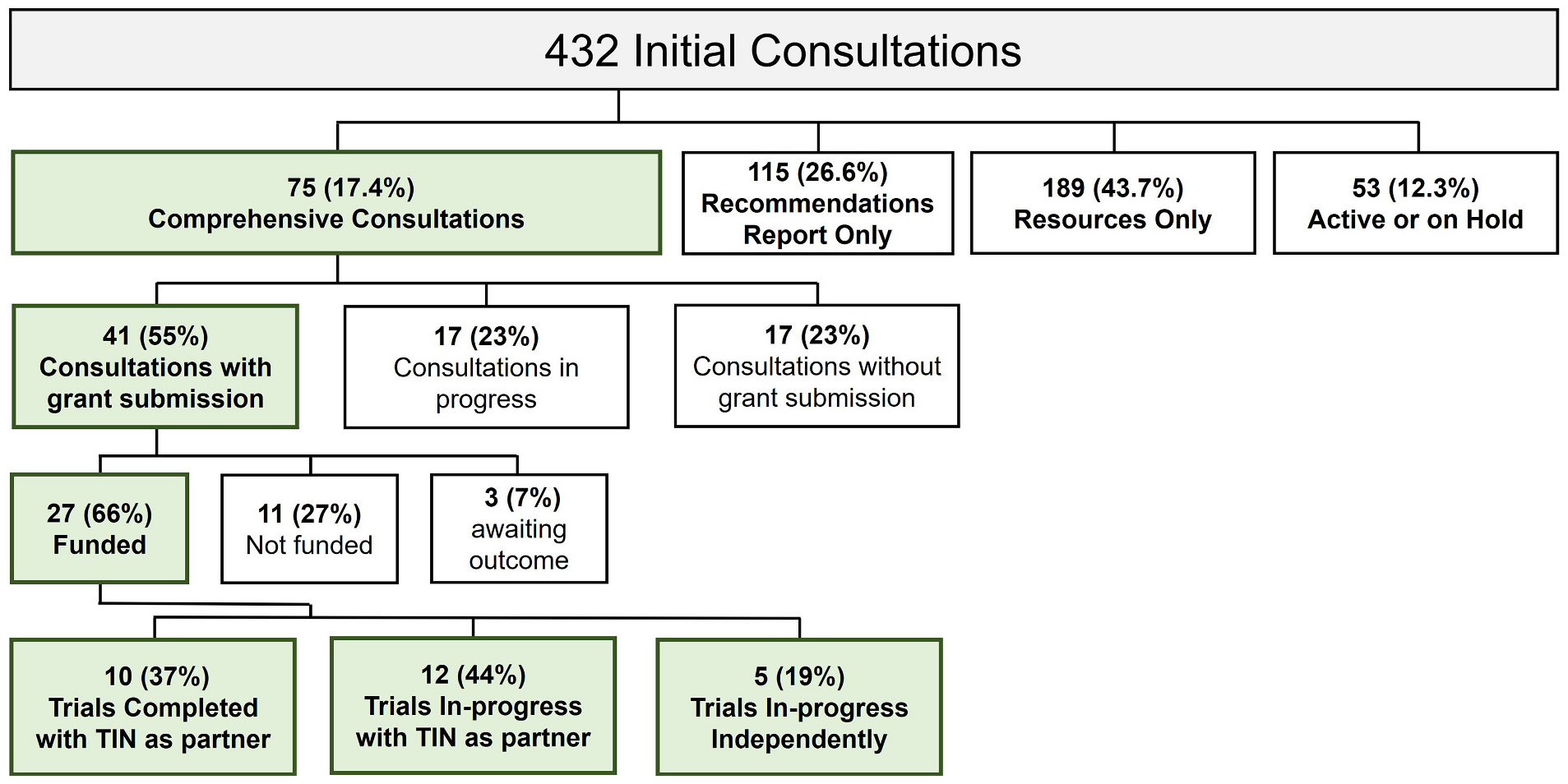
Results of the Comprehensive Consultation Process. Between October 2016 and June 2024, 432 proposals were assigned to a TIC/RIC for an initial consultation. The initial consultations resulted in either a comprehensive consultation, recommendations report only, resources only, or are active or on hold. Each of the seventy-five comprehensive consultations completed, received recommendations for each consultation. The comprehensive consultations resulted in various outcomes, including grant submission, consultations still in progress, or the consultation did not lead to a grant submission due to a sponsorship challenge outside the TIN’s scope, or the PI decided to not move forward without further preparation.

**Fig. 3.**
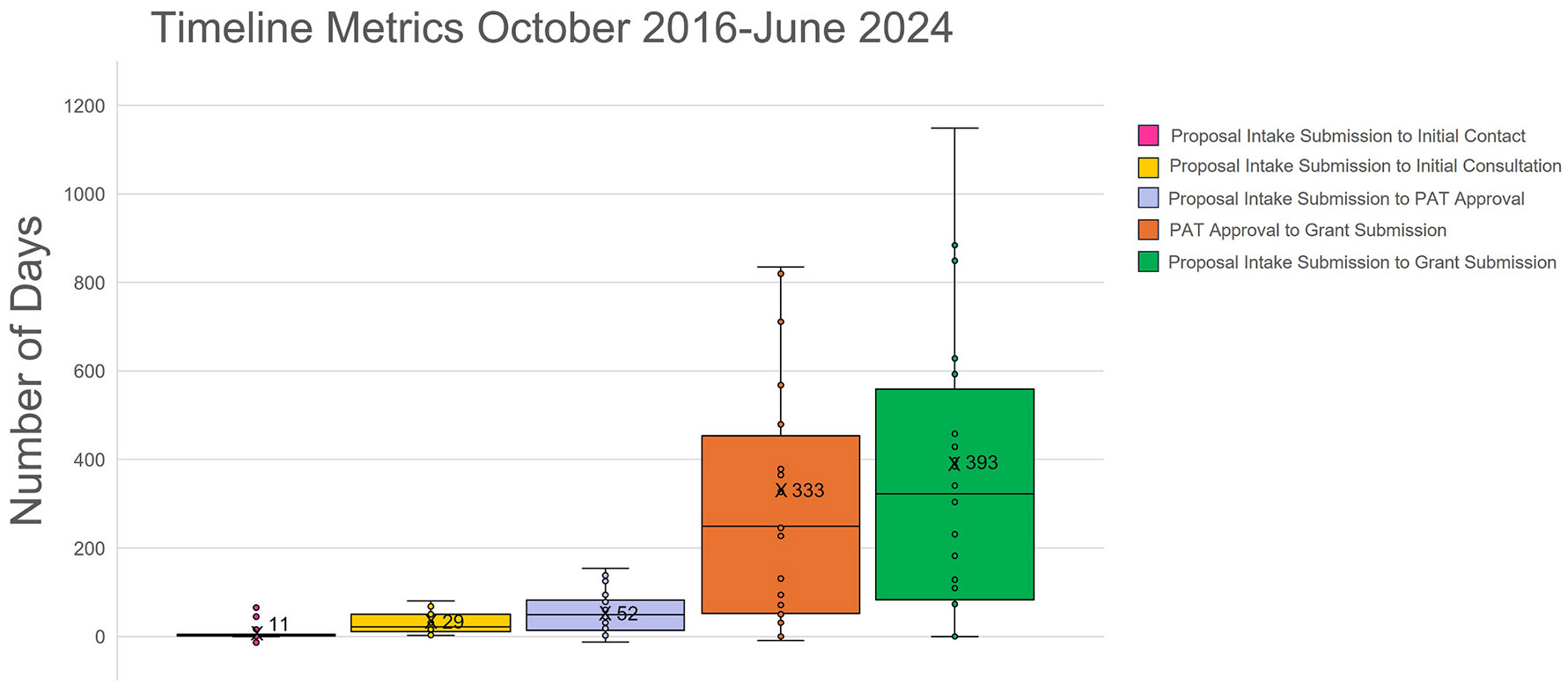
Box-and-Whisker Plot of the Consultation Timeline Metrics. Interval times in the Consultation process for the 41 grants that were submitted through the TIN. PAT approval to Grant submission, which includes the Comprehensive Consultation, and any necessary grant resubmissions, took an average of 333 days. The X on the box plots indicates the average.

From October 26, 2016, to June 1, 2024, 75 of the 432 Initial Consultations advanced to a Comprehensive Consultation (Fig. 2). Forty-one (55%) Comprehensive Consultations resulted in grant submissions, averaging 333 days from PAT approval to grant submission (Fig. 3). Of the 41 grant submissions, 27 (66%) were funded, of which 22 trials have utilized TIN coordinating centers as partnerships or engaged CTSA institutions as sites. Of the 75 total Comprehensive Consultations, 48 (64%) did not result in a funded grant. This includes 34 consultations (45%) that did not progress to submission at the time and 14 (19%) where an application was submitted but not funded at the time. Among the consultations that did not progress to submission, half remain in active Comprehensive Consultation, while the other 17 were discontinued due to factors such as challenges completing required pilot work, regulatory or logistical barriers related to obtaining biologics or meeting FDA requirements, investigator transitions, or external sponsorship issues beyond the TIN's scope. Of the 14 consultations that did lead to a grant submission but not funding, three applications are still under review. The remaining 11 were not awarded, primarily due to budget misalignment with study aims or feasibility concerns related to operational complexity or tight submission timelines.

## 4. Discussion

The TIN Comprehensive Consultation process facilitates the timely development of high-quality grant applications for the implementation of multicenter clinical trials within the CTSA consortium. Our data demonstrate a funding success rate of 66%, higher than the NIH success rate of research project grant applications received by the NIH in 2023 [1]. On average, the duration of the Comprehensive Consultation among TIN consultees was approximately 333 days. This includes delays such as timing requirements for primary grant submission, resubmissions at subsequent funding cycles, as well as other common and necessary delays inherent to the extramural funding process. These delays include waiting for sponsor-defined submission windows, responding to reviewer feedback, and, in some cases, changes in funding sponsor or funding mechanism as projects evolve or as investigators identify more appropriate opportunities. Compliance with sponsor evaluation processes reflect the TIN's commitment to partnering with consultees to develop well-designed, multicenter clinical trials.

These consultations use a structured, phased approach, and leverage experienced, nationwide resources so that investigators at any stage in their career can receive targeted, fully considered support for informative trial design and innovative operational execution at no cost to the consultee. Comprehensive Consultations have impactful and meritorious trials [12,13], embedding valuable innovations by the TIN [5,10,11]. The TIN Comprehensive Consultation model demonstrates a scalable, collaborative multi-institutional framework for enhancing the rigor and success of investigator-initiated multicenter trials. CTSAs and TIN provide a solution for more informative trials and generate new evidence to improve patient health.

## Data Availability

The datasets used and/or analyzed during the current study are available from the corresponding author on reasonable request.

## References

[1] Lane K, Palm ME, Marion E, , Approaches for enhancing the informativeness and quality of clinical trials: innovations and principles for implementing multicenter trials from the trial innovation network, J. Clin. Transl. Sci 7 (1) (2023) e131, 10.1017/cts.2023.560. 37396815 PMC10308427

[2] Lauer M, FY 2023 By the Numbers: Extramural Grant Investments in Research, National Institutes of Health, 2024. Accessed February 4, 2026. FY 2023 By the Numbers: Extramural Grant Investments in Research – NIH Extramural Nexus.

[3] Bernard GR, Harris PA, Pulley JM, , A collaborative, academic approach to optimizing the national clinical research infrastructure: the first year of the trial innovation network, J. Clin. Transl. Sci 2 (4) (2018) 187–192, 10.1017/cts.2018.319. 31011433 PMC6474372

[4] Harris PA, Dunsmore SE, Atkinson JC, , Leveraging the expertise of the CTSA program to increase the impact and efficiency of clinical trials, JAMA Netw. Open 6 (10) (2023) e2336470. Published 2023 Oct 2, 10.1001/jamanetworkopen.2023.36470. 37796498 PMC10773966

[5] Hillery S, Majkowski R, Wang Y, , Accelerating start-up cycles in investigator-initiated multicenter clinical trials, J. Clin. Transl. Sci 9 (1) (2025) e249, 10.1017/cts.2025.10180. 41395175 PMC12695510

[6] Harris PA, Wilkins CH, Lane K, , Enhancing Multicenter trials with the trial innovation network’s initial consultation process, JAMA Netw. Open 8 (5) (2025) e2512926, 10.1001/jamanetworkopen.2025.12926. 40440017 PMC12123472

[7] Harris PA, Kennedy N, Wilkins CH, , Insights from the trial innovation network’s initial consultation process, J. Clin. Transl. Sci 9 (1) (2025) e149. Published 2025 Jun 26, 10.1017/cts.2025.10084. 40735123 PMC12305380

[8] Wilkins CH, Edwards TL, Stroud M, , The recruitment innovation center: developing novel, person-centered strategies for clinical trial recruitment and retention, J. Clin. Transl. Sci 5 (1) (2021) e194, 10.1017/cts.2021.841. 34888064 PMC8634298

[9] Palm ME, Edwards TL, Wieber C, , Development, implementation, and dissemination of operational innovations across the trial innovation network, J. Clin. Transl. Sci 7 (1) (2023) e251, 10.1017/cts.2023.658. 38229905 PMC10790103

[10] Lane K, Hillery S, Majkowski R, , Selecting trial centers using a standardized, automated site assessment survey instrument (SASI), Contemp. Clin. Trials (2024), 10.1016/j.cct.2024.107583. Published online May 29. PMC11283960 38821259

[11] Lane K, Majkowski R, Gruber J, , Using gamification to enhance clinical trial start-up activities, J. Clin. Transl. Sci (2022) 1–22, 10.1017/cts.2022.405. PMC9274663 35836785

[12] Sullivan DJ, Gebo KA, Shoham S, , Early outpatient treatment for Covid-19 with convalescent plasma, N. Engl. J. Med 386 (18) (2022) 1700–1711, 10.1056/NEJMoa2119657. 35353960 PMC9006786

[13] Luciano M, Holubkov R, Williams MA, , Placebo-controlled effectiveness of idiopathic Normal pressure hydrocephalus shunting: a randomized pilot trial, Neurosurgery 92 (3) (2023) 481–489, 10.1227/neu.0000000000002225. 36700738 PMC9904195

